# GRIM-19: A Double-edged Sword that Regulates Anti-Tumor and Innate Immune Responses

**Published:** 2008-03-17

**Authors:** Shreeram C. Nallar, Sudhakar Kalakonda, Peng Sun, Dhan V. Kalvakolanu

**Affiliations:** 1 Department of Microbiology and Immunology, Marlene and Stewart Greenebaum Cancer Center, University of Maryland School of Medicine, Baltimore, MD 21201; 2 Molecular and Cellular Cancer Biology, Graduate Program in Life Sciences, University of Maryland School of Medicine, Baltimore, MD 21201

**Keywords:** cytokines, tumor suppression, apoptosis, transcriptional inhibition and immune response

## Abstract

Gene associated with retinoid-interferon-β-induced mortality (GRIM)—19, was originally identified as a critical regulatory protein necessary for Interferon-β-Retinoic acid-induced cell death. Overexpression of GRIM-19 activates cell death and its suppression or inactivation promotes cell growth. GRIM-19 targets multiple proteins/pathways for exerting growth control and cell death. However, GRIM-19 is also required for normal cellular processes. In addition, viruses ‘hijack’ GRIM-19 for their survival. Intracellular bacterial infections and bacterial products have been reported to induce the expression of GRIM-19. In this review, we will discuss the current status of GRIM-19 in growth control and innate immune response.

## Introduction

In multicellular organisms, temporal and spatial control of gene expression and gene products are very crucial for maintaining tissue homeostasis (Evan and Vousden, 2001). This balance comes essentially from the stochiometric and functional level(s) of various gene products that are involved in a process or/and necessary for such a cross talk. Any tilt in this delicately balanced process results in an abnormal cellular experience such as stress; and the cellular machinery tries to restore it to normalcy. Hence, higher order organisms have evolved multiple stress-sensing, alerting and relieving systems that are beneficial for organismal homeostasis. Cytokines are small-secreted proteins with multiple functions whose optimal responses are beneficial (Evan and Vousden, 2001). In this review, we will focus on a specific cytokine-induced novel growth-suppressor and its mechanisms of action.

## Pathways of Apoptosis

Apoptosis, a naturally-occurring programmed cell suicide mechanism, enables metazoans to control cell numbers in tissues; and eliminates cells that threaten the organism’s survival in an orderly manner (Kerr et al. 1994). There are at least two broad pathways that lead to apoptosis *viz*., extrinsic and intrinsic. In both pathways, signaling results in the activation of a family of cysteine proteases, named Caspases, which act in a orchestrated proteolytic machinery to dismantle the cell architecture (Czerski and Nunez, 2004). In the extrinsic pathway, extra-cellular protein(s) trigger the apoptotic response, while in the intrinsic pathway intra-cellular factor(s) elicit the apoptotic response. The extrinsic pathway depends on cell surface receptor systems and the intrinsic pathway is activated by oncogenes, DNA damage, hypoxia, mitochondrial damage *etc*.

Receptor-mediated apoptosis involves specific ligand-receptor interactions that transduce signals which culminate in the activation of distinct Caspases. Eight such death receptors (DRs) have been characterized so far: DR1 (also known as TNFR1, CD120a, p55 and p60) (Sutheesophon et al. 2005), DR2 (also known as FasR, APO1 and CD95) (Jung and Kim, 2004), DR3 (also known as APO3, LARD, TRAMP and WSL1) (Shi et al. 2003), DR4 (also known as APO2, TRAILR1) (Kaufmann and Steensma, 2005; Luschen et al. 2005), DR5 (also known as TRAILR2, KILLER and TRICK2) (Kaufmann and Steensma, 2005; Luschen et al. 2005), DR6 (Shi et al. 2003), EDAR (Ectodysplasin-A Receptor) and NGFR (Nerve Growth Factor Receptor) (Wajant, 2003). These receptors are distinguished by a cytoplasmic region, of approximately 80 amino acid residues, termed the death domain (DD). Upon activation, a number of proteins are recruited to the DD and subsequently a signaling cascade is activated. Two such DR-signaling complexes can be distinguished: the 1^st^ group composed of the DISCs (Death-Inducing Signaling Complexes) that are formed with DR2, DR4 and DR5 have similar composition and the 2^nd^ group composed of TNFR1, DR3, DR6 and EDAR that have different set of molecules which transduce the signal leading to activation of the initiator Caspase (pro-Caspase8 to Caspase8 through self-cleavage) which activates down-stream Caspases. However, the latter group is known to transduce survival signals (Fas et al. 2006; Sandu et al. 2005).

In addition to receptor-mediated apoptosis, there is another pathway activated by various forms of cytotoxic stress-inducers like γ–rays, *uv*-rays, drugs like Actinomycin-D, removal of cytokines *etc* that involves altering the mitochondrial permeability transition (Harris and Levine, 2005) and subsequent release of cytochrome-c and formation of the Apoptosome, a catalytic multi-protein platform that activates Caspase9, which in turn activates Caspase3 leading to the later events involved in cell death (Pirnia et al. 2002).

The intrinsic pathway is activated when an injury occurs within a cell such as DNA damage, hypoxia etc. The p53 protein is a sensor of cellular stress and is a critical activator of the intrinsic pathway (Haupt et al. 2003). Activated p53 initiates apoptosis by activating pro-apoptotic Bcl-2 family members while repressing the expression of anti-apoptotic Bcl-2 members and cellular inhibitors of apoptosis proteins (CIAPs). Other p53 targets include BAX, Noxa, PUMA, BID and genes which cause an increase in ROS (reactive oxygen species) and damage mitochondrial components (Uren et al. 2005).

Most of the above pathways culminate in the activation of Caspase-activated DNAse (CAD) (Uren et al. 2005), generation of pro-apoptotic peptides from the Bcl-2, Bcl-X_L_, Bid proteins. Although many death signals employ at least one of these pathways in later stages of cell death, their upstream factors are quite often different.

## Interferon Signaling in Growth Control

Interferons (IFNs) are critical regulators of anti-viral, anti-microbial, anti-tumor, cellular differentiation and immune response activities (Boehm et al. 1997; Kalvakolanu, 2000; Sen, 2000). The primary IFN-signaling is routed through the JAK-STAT pathway (Levy and Darnell, 2002; Stark et al. 1998) and the biological responses of IFNs is controlled by two classes of transcription factors *viz*., Interferon-gene Regulatory Factors (IRFs) and STATs (Fujita et al. 1989; Pine et al. 1990). The former regulate IFN genes and some ISGs while the latter primarily regulate ISGs. These factors cross-regulate each other and form complexes with other factors to deliver distinct biological response(s) based on recruitment to different promoter elements. The Interferon-Stimulated Response Element (ISRE) is one such element where a multimeric transcription factor (ISGF3) comprising of IRF9, STAT1 and STAT2 binds and drives transcription of certain ISGs (Levy and Darnell, 2002; Stark et al. 1998). In a similar manner, binding of activated STAT1 following IFN-γ treatment drives transcription of some ISGs that possess a Gamma-Activated Sites (GAS) element in their promoter (Shuai et al. 1994; Shuai et al. 1992). Cytokine-induced STAT-driven responses are controlled by feedback mechanisms that control the time/extent/amplification from the initial signaling event. The first mechanism involves phosphatases that dephosphorylate active STAT proteins *e.g.* dephosphorylation of STAT1 and STAT5 by the phosphatase TcPTP (Aoki and Matsuda, 2002; ten Hoeve et al. 2002). The second mechanism involves inhibitors that bind to activated STAT proteins, which prevent their DNA binding e.g. the binding of PIAS1 to STAT1 (Chung et al. 1997; Liu et al. 1998). The third mechanism involves STAT-induced factors called Suppressor of Cytokine Signaling (SOCS) proteins that bind to the JAKs and inhibit their activity *e.g.* SOCS3 against STAT3 (Hanada et al. 2003; Karlsen et al. 2001; Morita et al. 2000; Starr and Hilton, 1999). Multiple SOCS proteins have been described that target distinct JAKs (Alexander et al. 1999; Croker et al. 2003).

IFNs were the first cytokine therapeutics used clinically for the therapy of many viral diseases, malignancies or syndromes (Borden et al. 2000). As expected, IFNs, IFN receptors and the transcription factors regulated by them play important roles in eliminating neoplastic growth (Gresser and Belardelli, 2002; Ikeda et al. 2002). For example, mutations in the human IRF1 gene has been linked to myeloid leukemia (Willman et al. 1993); deletion of the IRF8 gene causes a CML-like disease in mice (Holtschke et al. 1996), while in humans expression of IRF8 was depressed (Schmidt et al. 1998), and in CML patients having the BCR-ABL oncogene re-expression of IRF8 suppressed the myelo-proliferative disorder (Hao and Ren, 2000). The IFN-γ receptor^−/−^ and STAT1^−/−^ mice are highly susceptible to chemical carcinogenesis (Kaplan et al. 1998). Although a great deal is known about anti-viral actions of IFNs, their anti-tumor actions are not fully understood. The anti-tumor activity of IFNs as a single agent is comparable to that of many therapeutics. In some cases, insensitivity to IFN-induced anti-tumor effects could be overcome by combining with other agents such as the cell differentiating agents, retinoids.

## Retinoid Signaling in Growth Control

Retinoids are a group of Vitamin-A-related compounds that regulate cell growth, differentiation and embryogenesis in vertebrates (Love and Gudas, 1994). All *trans* retinoic acid (ATRA or RA), a closest metabolite of Vitamin A, can drive all functions except vision (Love and Gudas, 1994). The importance of retinoids in growth regulation is highlighted by a higher incidence of cancers in animals deprived of Vitamin A that can be reversed or controlled by RA treatment (Bjelke, 1975; Kuwata et al. 2000). The biological actions of retinoids are exerted *via* two structurally similar, but genetically distinct, nuclear retinoid receptors *viz*., Retinoic Acid Receptors (RARs) and Retinoid X Receptors (RXRs) that are constitutively bound to specific DNA sequence motifs known as the Retinoic Acid Response Elements (RARE), as heterodimers (Chambon, 1996; Kliewer et al. 1992; Mangelsdorf and Evans, 1995; Nagpal et al. 1992). A number of isotypes of RARs and RXRs (α, β and γ) and their subtypes participate in retinoid-induced transcription. Most RAR and RXR genes are constitutively expressed except for the RARβ gene whose expression is up-regulated by RA-treatment (Chambon, 1996; Mangelsdorf and Evans, 1995). Mutations in the RAR and RXR genes occur rarely. The expression of RARβ gene is inhibited in some cancers and its re-expression restores growth arrest in response to RA (Liu et al. 1996). Indeed transgenic mice expressing anti-sense RARβ gene develop lung cancers; although the exact mechanism(s) are unknown (Berard et al. 1996). The only well-defined mutation that involves RARs is a reciprocal translocation that occurs between the RARα (human chromosome 17) and the promyelocytic leukemia (human chromosome 15) loci in some forms of acute promyelocytic leukemia (APL) (de The et al. 1991; Kakizuka et al. 1991). Despite this translocation, PML-RARα protein responds to RA treatment and leukemic cell growth is successfully controlled in APL patients (Chomienne et al. 1990; Hofmann, 1992). The PML gene is induced by IFNs *via* STAT1-dependent pathway and implicated in the anti-viral actions of IFNs (Pelicano et al. 1997; Regad et al. 2001).

## Crosstalk Between IFNs and Retinoids

Although IFNs and retinoids induce genes using different signaling pathways, several studies have shown that a crosstalk exists between their growth-suppressive pathways (Chelbi-Alix et al. 1998; Nason-Burchenal et al. 1996; Ruggero et al. 2000). For example, RA-induced expression of STAT1 augments IFN-induced responses while a mutant STAT1 inhibits RA-induced differentiation (Dimberg et al. 2000; Gianni et al. 1997; Kolla et al. 1997; Matikainen et al. 1997). Experimental and clinical models have shown that IFN-RA combination synergistically inhibits tumor growth and tumor angiogenesis compared to either agent alone (Frey et al. 1991; Hemmi and Breitman, 1987; Lindner et al. 1997; Marth et al. 1986). Furthermore, apoptosis occurred in cells that lacked either a functional p53 or/and Caspase3 implying the factors are distinct from known death regulators. Using a genetic screen, our lab isolated the mediators of the growth-suppressive effect of IFN/RA. These were collectively called the GRIMs (Genes associated with Retinoid-Interferon-induced Mortality).

## Isolation of the GRIMs

The use of genetic approaches to identify genes based on their function will rapidly advance our understanding of cellular processes. Employing an anti-sense technical knockout strategy (Deiss and Kimchi, 1991), we isolated genes that regulate cell death in response to IFN/RA treatment (Hofmann et al. 1998). Briefly, total RNA prepared from untreated and IFN/RA-treated cells (multiple time points) were pooled and a cDNA library was constructed. This unamplified cDNA library was cloned into an episomal vector pTKO1 to generate an anti-sense library. When transfected into cells and selection pressure applied with IFN/RA, cells that harbor an anti-sense against a death-associated gene transcript will selectively survive in the presence of IFN/RA. Individual episomes were rescued from the surviving colonies and tested for protection against IFN/RA-induced cell death to eliminate false positives. Episomes that consistently conferred protection against IFN/RA-induced cell death were collectively called the GRIMs. Partial sequences have revealed that most of these genes were novel. Isolation of several GRIMs using this approach suggests the existence of a coordinated network of factors that are necessary for executing IFN/RA-induced cell death. Consequently, interfering with one gene product is sufficient to abolish cell death. In this review, we will focus on GRIM-19, which has emerged as a key player in growth control and apoptosis using *in-vitro* and *in-vivo* models.

## GRIM-19—An Introduction

In humans, the gene for GRIM-19 maps to a locus on chromosome 19 (Chidambaram et al. 2000), whose loss has been implicated in prostate cancer progression (Gao et al. 1999). The gene is ~13.0 Kb and consists of 5 exons. Other salient features of this gene are: a) extremely GC-rich, b) the first intron is very long ~9.0 kbp, c) minimal 5′ and 3′ UTRs. The protein encoded by this gene has a mass of ~16 kDa with no apparent sequence-motif or domain identities with other proteins in the databases. Immunohistochemical studies revealed that GRIM-19 is distributed in the cytoplasm and nucleus as punctate structures indicative of its presence in certain protein complexes *in-vivo* (Angell et al. 2000). Indeed, a subsequent study reported GRIM-19 to be a part of purified Complex-I of the mitochondrial electron transport system (ETS) that generates the electro-chemical gradient required for ATP synthesis (Fearnley et al. 2001; Murray et al. 2003). This characteristic may indicate as to why a homozygous deletion results in embryonic lethality in mice. However, studies that are more detailed are required for deciphering the actual role of GRIM-19 in steady-state. Ectopic expression of GRIM-19 induces cell death and moderate levels of it sensitizes cells to IFN/RA-induced apoptosis (Angell et al. 2000).

## Viruses Target GRIM-19 for Cell Survival

The initial anti-GRIM-19 activities came from studies on human Herpesvirus-8 (HHV-8) that is frequently associated with Kaposi’s sarcoma (KS), B-cell Lymphoma (BCL), multicentric castleman disease (MCD) and primary effusion lymphoma (PEL) (Gillison and Ambinder, 1997; Neipel and Fleckenstein, 1999). The viral genome, in addition to several structural proteins, also codes for a number of proteins homologous to various cellular proteins *viz*., viral IL-6 (vIL-6), viral Bcl2 (vBcl2), and three viral homologues of IRFs (vIRFs) etc. The viral IRFs (vIRFs) are implicated in cellular transformation and also function as arsenals to counter the host response against invading pathogen (Burysek and Pitha, 2001; Moore et al. 1996). The vIRF1 protein inhibits IFN-gene expression by interfering with the interaction of CBP/p300 with co-activator, IRF3 (Burysek et al. 1999; Li et al. 2000; Lin et al. 2001). It does not bind to DNA like IRFs. We have shown that vIRF1 binds with GRIM-19 and blocks its ability to induce apoptosis (Seo et al. 2002). However, vIRF1 also activates the expression of growth promoting transcription factor c-Myc (Jayachandra et al. 1999). The vIRF2 protein inhibits protein kinase R (PKR), a translation-inhibitory kinase induced by type-I IFNs and double-stranded RNA, that phosphorylates translation initiation factor (eIF2α) leading to inhibition of protein synthesis (Burysek and Pitha, 2001). The human Papillomavirus (HPV) E6 protein of the high-risk strains, but not the low-risk strains, binds to GRIM-19 (Seo et al. 2002). Other viruses appear to target GRIM-19 using different mechanisms. For example, upon Vaccinia virus (VV) infection, *de-novo* levels of GRIM-19 dropped significantly at 2 h post-infection and maintained at low level as long as 16 hours post-infection (Guerra et al. 2003). The factors/mechanisms involved in this process are not identified. A more provoking mechanism is employed by human Cytomegalovirus (CMV). The viral genome encodes a highly abundant 2.7-kb RNA transcript (β2.7) that accounts for 20% of total viral transcription (Greenaway and Wilkinson, 1987; Spector, 1996) of unknown function and no protein product has been detected in infected cells suggesting that it may function as a non-coding RNA (McSharry et al. 2003). The virus employs this RNA to ‘lockup’ GRIM-19 in Complex-I, hence renders it ineffective in triggering the apoptotic cascade (Reeves et al. 2007). A viral mutant, lacking β2.7, was ineffective at preventing apoptosis upon infection. The functionality of β2.7 did not change when provided *in trans* i.e. in an expression vector, suggesting its independence from other viral proteins. Inspite of the virus up-regulating steady-state levels of mRNAs coding for some subunit proteins in the Complexes-I to V, GRIM-19 levels were unaffected as long as 120 hours post-infection (Reeves et al. 2007). Why and how the virus achieves this state is worth investigating. Reeves et al. (2007) also suggested that localization of GRIM-19 is not constant and can change according to the need of the cell, which may explain the disparity among research groups about the localization of GRIM-19 (more in later sections).

## Cellular Targets of GRIM-19

To gain a better understanding of GRIM-19-induced growth suppression, we had used yeast two-hybrid (Y2H) screen to identify the cellular targets. Initial studies identified STAT3 as one of its interacting partners (Lufei et al. 2003; Zhang et al. 2003). STAT3 is central to several diverse biological functions in mammals including cytokine-driven responses, differentiation, embryonic growth and cell survival (Akira et al. 1994; Bowman et al. 2000; Zhong et al. 1994). In normal cells, STAT3 activity is tightly regulated by feedback inhibitory mechanisms that prevent excessive signaling. Constitutive activation of STAT3 has been documented in many tumors and it inhibits apoptosis (Bowman et al. 2000; Bromberg, 2001; Cattaneo et al. 1998; Garcia et al. 1997; Gouilleux-Gruart et al. 1996; Schrell et al. 1998). Interference with STAT3 function promotes apoptosis (Catlett-Falcone et al. 1999; Epling-Burnette et al. 2001; Gao et al. 2005; Grandis et al. 2000; Nielsen et al. 1999; Niu et al. 2001). Interestingly, GRIM-19 interferes with STAT3 distinctly and does not fall into the known categories of cytokine-induced feedback regulatory mechanisms. Our results have indicated that GRIM-19 binds to the trans-activating domain (TAD) of STAT3 and blocks its activity. Lufei et al. (2003) reported that GRIM-19 binds to the coiled-coil, DNA-binding and linker domains of STAT3. This study mainly used overexpression strategy. In contrast, our results were inferred using point mutations in the TAD and STAT3 truncations in a *stat3*^−/−^ background. We have also shown that TAD alone is insufficient for GRIM-19 binding suggesting an additional site or/and factor(s) mediating this interaction (Zhang et al. 2003). Lastly, we have recently reported the effects of GRIM-19 binding to a constitutively active form of *stat3* (S3C) (Kalakonda et al. 2007b). S3C protein has been engineered to behave like phosphorylated-STAT3 *i.e*. spontaneous homo-dimer formation, that localizes it to the nucleus (Bromberg et al. 1999). By Western analyses, we have shown S3C-GRIM-19 complexes in the nuclear extracts, which is contrary to the mechanism suggested by Lufei et al. (2003) i.e. GRIM-19 functioned only to block the nuclear entry of activated STAT3. Additionally, binding of GRIM-19 to STAT3 or S3C did not prevent the DNA-binding of STAT3 or S3C as revealed by ChIP analysis of STAT3-responsive gene promoters, which again is contradictory to the mechanism suggested by Lufei et al. (2003) wherein GRIM-19 blocked the DNA-binding ability of STAT3 by steric hindrance to its DNA-binding domain. It should be noted that Lufei et al.’s (2003) conclusions were entirely drawn on the basis of overexpression of STAT3 and GRIM-19 in COS cells. It is likely that overexpression-associated artifacts may explain the hindrance of nuclear translocation of STAT3. Despite these differences with respect to localization of GRIM-19, mechanism of action, intra-cellular environment of complex formation *etc*, all studies agree on the death-inducing potential of GRIM-19 and its anti-STAT3 effects. Is anti-STAT3 activity just sufficient to block cell proliferation *vis-à-vis* tumor growth in all scenarios? Our recent studies have shown that interference with STAT3 function either by direct blockade of STAT3 expression, by intratumoral administration of shRNA expression vectors (Zhang et al. 2007) or by intratumoral expression of GRIM-19 (Zhang et al. Clin Cancer Res, In press) is one major mechanism of tumor growth suppression *in vivo*.

Another group reported a growth-promoting gene product that interacts with GRIM-19 using Y2H screen (Zhang et al. 2004). This gene, *gw112*, bears little homology to other known genes and is highly expressed in the tumors of the gastro-intestinal tract. Immunochemical analysis revealed that most of GW112 protein to be in mitochondria and some in the nucleus as distinctive foci. Levels of GW112 were very high during early S-phase that gradually declined over late S-phase and was required for G_2_/M transition (Kobayashi et al. 2007). However, reduction of GW112 levels by siRNA-based strategy resulted only in a slight increase in apoptosis, which is drastically different compared with STAT3 interference. Overexpression of GW112 can attenuate H_2_O_2_ and IFN/RA-induced apoptosis (Kobayashi et al. 2007). Although the exact function and growth-promoting mechanism of GW112 is not clear, three genes products involved in apoptosis and oxidative-stress (PIG12, GADD153 and c-Abl) were down-regulated in GW112-overexpressing cells, while the same genes were up-regulated in GRIM-19-overexpressing cells or/and in response to IFN/RA treatment (Kobayashi et al. 2007).

Barnich et al. (2005) identified NOD2 (nucleotide-binding oligomerization domain containing 2) as an interacting partner of GRIM-19 in another Y2H screen. NOD proteins are a group of intra-cellular bacterial molecular pattern-recognition sensors that are thought to utilize RIP2/RICK and induce NF-*κ*B activation. Together with cell membrane-associated TLRs (Toll-Like Receptors), NOD proteins play important roles in innate and acquired immunity. However, the study found GRIM-19 to be crucial for NF-*κ*B activation following NOD2-mediated recognition of bacterial muramyl dipeptide. GRIM-19 appeared to be a key component in NOD2-mediated innate mucosal responses. Interestingly, GRIM-19 localized in vesicles, which are not near the cell membrane, not in mitochondria or the nucleus in this study whose significance needs to be addressed. Barnich et al. (2005) also reported up-regulation of GRIM-19 when challenged by intra-cellular bacteria.

One of the recent GRIM-19 binding partners is HtrA2, a serine protease localized in the mitochondria (Ma et al. 2007). In mammalian cells, inhibitors of apoptosis proteins (IAP) block Caspase activation. One such protein, X-linked IAP (XIAP) inhibits Caspase9. HtrA2 degrades XIAP to relieve the inhibition on Caspases. Upon IFN/RA treatment, HtrA2 and GRIM-19 are released from mitochondria and they form a complex in the cytosol that degrades XIAP. The interaction between GRIM-19 and HtrA2 was significantly stronger upon IFN/RA treatment suggesting some post-translational change(s) that increases the binding potential (Ma et al. 2007). Indeed, a recent study reported dynamic phosphorylation of Threonine 113 of GRIM-19 in steady-state (Palmisano et al. 2007). Interestingly, one of the GRIM-19 point mutations, (Arginine 115 Proline) reported in the thyroid cancer (Maximo et al. 2005), is in close vicinity to the phosphorylation site. Whether such phosphorylation has any biological relevance needs to be experimentally defined. These in conjunction with our earlier finding that the deletion of the C-terminus of GRIM-19 abrogates its death-promoting function (Angell et al. 2000) appear to be relevant in this context while the full spectrum of changes that should occur to GRIM-19 for it to induce apoptosis cannot be addressed at present.

Our latest report addressed the *src*-family of tyrosine kinases, which are important regulators of various cell growth responses *viz*., cellular transformation, altering transcription, cytoskeletal properties (Behrens et al. 1993; Frame, 2004; Frame et al. 2002; Lin et al. 1983; Perucho and Esteban, 1985) etc. Since STAT3 and Src both are pro-growth genes and having studied the effect of GRIM-19 on STAT3, we addressed the role of GRIM-19 on Src. GRIM-19 suppressed Src-induced cellular transformation *in vitro* and *in vivo* by down-regulating the expression of a number of STAT3-responsive genes (Kalakonda et al. 2007a). In addition, GRIM-19 inhibited the Src-induced cell motility and metastasis by suppressing the tyrosyl-phosphorylation of focal adhesion kinase (FAK), paxillin, E-cadherin and gamma-catenin. The effects of GRIM-19 on Src-induced cellular transformation were reversible in the presence of GRIM-19-specific short hairpin RNA, indicating its direct effect on transformation. GRIM-19-mediated inhibition of the Src-induced tyrosyl phosphorylation of cellular proteins, such as FAK and paxillin, seemed to occur independently of the STAT3 protein. GRIM-19 had no significant effect on the cellular transformation induced by other oncogenes such as *myc* and *Ha-ras* (Kalakonda et al. 2007a). Thus, GRIM-19 not only blocked Src-induced gene expression through STAT3 but also the activation of cell adhesion and motility-related molecules. Similar effect on cell motility is controlled by another protein Stathmin that destabilizes microtubules (Ng et al. 2006). STAT3 interacted with Stathmin thus antagonizing its destabilizing activity. Since GRIM-19 interacts with STAT3, these inhibitory networks may be operative in parallel.

Another interesting report concerns the role of Janus tyrosine kinase, Tyk2, in mitochondrial respiration in primary pro-B lymphocytes in a mouse model (Potla et al. 2006). Although *tyk2*^−/−^ mice were phenotypically normal, cells from such mice exhibited abnormal responses to inflammatory challenges in different cell types; specifically pro-B cells were markedly deficient in basal oxygen consumption and exhibited a significant decrease in steady-state cellular ATP levels which were attributed to impaired complex-I, complex-III and complex-IV functions of the mitochondrial ETS. However, Tyk2 was required for maintenance of both complex-I-dependent respiration as well as induction of apoptosis in cells incubated with IFN-β. Consistent with the role of Tyk2 in the regulation of tyrosyl-phosphorylation of STAT3, expression of constitutively active STAT3 (S3C) could restore the mitochondrial respiration and IFN-β-stimulated apoptosis in Tyk2^−/−^ cells treated with IFN-β. Though S3C did restore IFN-β-stimulated apoptosis, it was incomplete suggesting another Tyk2-dependent pathway is necessary for the full response. This appears to be an enigma wherein STAT3 phosphorylation stabilizes complex-I respiration, Tyk2 and phosphorylated STAT3 are required for apoptosis, the same JAK-STAT pathway induces GRIM-19 while the receptor stimulation causes GRIM-19 release from complex-I. It is debatable as to which route of signaling is stronger to achieve the outcome *i.e.* survival/apoptosis. These results could also be interpreted as a novel mechanism to control STAT3 activity wherein, GRIM-19 from some of the complex-I gets released to bind STAT3 and control the temporal or/and spatial activity of STAT3 in the nucleus. However, novel roles for non-phosphorylated STAT3 have been reported (Yang et al. 2005) suggesting other factors or/and promoter-context may also be important. More importantly, the generality of this observation to other cell types, other than pro-B cells, needs to be verified.

## Inducers of GRIM-19

As mentioned above, coupling of TLR4 and NOD2 signals is necessary for innate immunity. Does activation of cell membrane-associated TLRs result in an up-regulation of GRIM-19? When CaCo2 cells were challenged with *Salmonella typhimurium*, *grim-19* mRNA levels were 2.26-fold higher, within 2 hours, than control cells whilst a challenge with non-pathogenic *E. coli* did not induce the mRNA levels significantly (Barnich et al. 2005). Similarly, BCG-CWS up-regulated GRIM-19 levels in monocytes and DCs prepared from peripheral blood (Begum et al. 2004) whereas only a live periodontal pathogen (*Porphyromonas gingivalis*) could up-regulate GRIM-19 levels, neither its LPS nor its fimbrial proteins, in peripheral blood monocytes (Zhou and Amar, 2006). The kinetics of GRIM-19 up-regulation in CaCo2 cells is very rapid compared to stimulation by BCG-CWS or *P. gingivalis* or IFN/RA. Eventhough this variability could be a cell-specific effect or the specificity of Ligand-TLR interaction, the mechanism or/and pathways involved merits investigation. On closer examination, one may interpret these results as: 1) the actual cells that encounter the pathogen i.e. natural route of entry, can up-regulate apoptotic machinery very rapidly to control the infection; 2) cells of the immune system have some time-lag in up-regulating GRIM-19; and 3) a signal from the initial event invokes cells of the immune system to respond to this signal and up-regulate other defense mechanisms, one of which involves GRIM-19. Although there are reports of *E. coli* LPS being more potent than, *P. gingivalis* LPS, the latter has been known to modulate the host response to facilitate colonization (Agarwal et al. 1995; Darveau et al. 2002; Murray and Wilton, 2003; Reife et al. 1995), while *E. coli* being a common intestinal flora is not recognized as a pathogen by the intestinal cells or cell line. In an analogous system, differential-response and enrichment of GRIM-19 ESTs was observed in hemocytes from mosquitoes, *Armigeres subalbatus and Aedes aegypti*, challenged with *E. coli* and *Micrococcus luteus* (Bartholomay et al. 2004).

## Patho-Physiological Relevance of GRIM-19

In this section, we will discuss the role of GRIM-19 in different pathogenic processes. Chronic Obstructive Pulmonary Disease (COPD) is a collective term for describing two distinct lung diseases: emphysema and chronic bronchitis, caused by cigarette smoking (Petty, 2002) that accounts for 80%–90% of COPD cases in the United States (Sethi and Rochester, 2000). However, only 15%–20% of heavy smokers develop clinically significant airflow obstruction, which suggests a genotypic influence in disease development. The genes that determine susceptibility through disease progression are poorly understood (Mayer and Newman, 2001). Employing SAGE, Microarrays and qRT-PCR, (Ning et al. 2004) showed that *grim*-*19* mRNA levels were higher in smoker’s lung compared to non-smokers, in addition to *cdkn1a* and *cdc2l1*. However, mitochondrial ETS complex-I activity was reduced by 50%.

Apoptosis-inducing factor (AIF) is a mitochondrial flavoprotein that, after apoptosis induction, translocates to the nucleus where it participates in apoptotic chromatinolysis (Susin et al. 1999; Ye et al. 2002; Yu et al. 2002). The physiological role of AIF in the mitochondria is not clear at this stage. Though AIF is not a part of sub-complexes of complex-I or mature complex-I or any super-complexes, it is required for proper assembly and/or maintenance of respiratory chain complex-I. AIF knockdown resulted in decreased GRIM-19 (NDUFA13), NDUFB6, NDUFS7 and NDUFA9 levels, all of which are parts of complex-I (Vahsen et al. 2004). However, AIF^−/−^ cells still had complete complex-I but in reduced abundance resulting in lower enzymatic activities and metabolized glucose like cancer cells.

In a study of patients with inflammatory bowel disease (IBD) *viz.*, Crohn disease (CD) and Ulcerative Colitis (UC), Barnich et al. (2005) showed significantly lower abundance of GRIM-19 in the involved areas compared to the non-involved areas of the intestinal mucosa. The mechanism(s) of such down-regulation is/are not known. This may be a very good working model for accessing the role of GRIM-19 in the development of neoplasia and metastasis.

In a very recent collaborative study we have shown that, focal cerebral ischemia up-regulated GRIM-19 levels significantly in the affected areas of the brain in a rat model. Upon treatment of neuroblastoma cell line with IFN/RA for 48 h, GRIM-19 levels were up by ~15-fold, while focal ischemia for 24 h could up-regulate *grim-19* mRNA ~32-fold but only 50% increase in protein level (Mehrabian et al. 2007). This discordance between protein and RNA levels could be due to an inhibition of protein synthesis in ischemically damaged cells. However, a cell-specific effect was also evident with respect to *grim-19* induction by hypoxia; the ipsilateral lobes had higher GRIM-19 levels compared to the contralateral lobes and the neurons had higher GRIM-19 levels compared to astrocytes.

## GRIM-19 and Cancer

The full development of cancer involves multiple genetic changes that occur in a tumor-specific manner. Mutations/repression/loss of genes coding for transcription factors and apoptotic machinery have been implicated in tumor growth. As mentioned earlier, viruses and other cellular factors bind to GRIM-19 and prevent its death-activating function. Recently, mutations in the human *grim-19* coding region were reported in Hürthle cell thyroid carcinomas (Maximo et al. 2005). To gain better understanding of molecular changes that appear in primary renal cell carcinoma (RCC), we compared the proteomes of normal kidney and RCC (Alchanati et al. 2006). Unlike prostate tumors, chromosome 19 anomalies are extremely rare in RCC. Results from this study revealed that: 1) 93% of RCCs lost GRIM-19 expression, and in the remainder only weak expression was noted, (2) Loss of GRIM-19 appears to be a characteristic of clear cell type RCCs and (3) a low frequency loss of GRIM-19 also occurs in prostatic and transition cell carcinomas. In most cases, tumor-derived GRIM-19 mRNA seems to harbor no mutations, except for a conservative substitution in one case (Alchanati et al. 2006). In those cases where complete loss of mRNA occurred, no gross deletions in the GRIM-19 gene were observed as analyzed by genomic PCR of tumor and normal DNAs (Nallar and Kalvakolanu, unpublished observations). Methylation or/and loss of transcription factor engagement with the promoter could be the reasons for loss of expression.

We have also profiled a few oral squamous cell carcinoma (SCC) samples where the levels of GRIM-19 was not significantly different, except for one sample that had a complete loss of expression, but STAT3 levels were significantly higher in the tumors of matched samples (Nallar and Kalvakolanu, unpublished). An earlier study by (Nindl et al. 2006) on cutaneous SCC, by microarray expression profiling, concluded no significant differences with respect to GRIM-19 levels. A recent study by (Gong et al. 2007) on colorectal carcinomas reported higher levels of STAT3 and lower levels of GRIM-19 in matched samples. No mutation was detected in GRIM-19 gene in the 23 specimens studied in this study.

## Conclusions

IFNs have been on the forefront in basic and clinical investigations and have emerged as a potent therapeutic agent; however, the least understood mechanism of IFN action is regulation of cell growth control. In this review we described the actions of an IFN/RA-induced gene, GRIM-19, in growth control. Although the GRIMs were identified as IFN/RA-induced cell death regulators *prima facie*, they may participate in growth- regulatory processes controlled by other ligands and in other physiologic processes in normal cells (Table 1). Indeed, recent studies have shown a) GRIM-19 to be a part of the membrane module of complex-I, that is added during the pre-final stage of complex-I assembly (Vogel et al. 2007) and b) GRIM-19 is essential for Ca^++^ homeostasis during embryogenesis (Chen et al. 2007).

**Figure 1:**
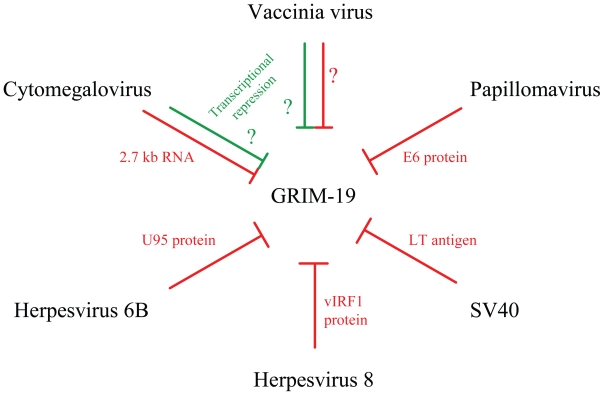
Viral Inhibitors of GRIM-19. Viruses effectively block GRIM-19’s action either by preventing its release from mitochondria and/or its action by sequestering with viral gene products. Lines in Red indicate blockade of GRIM-19 action involving the indicated viral components. Lines in Green indicate transcriptional repression. Mechanism of transcriptional repression is not known.

**Figure 2:**
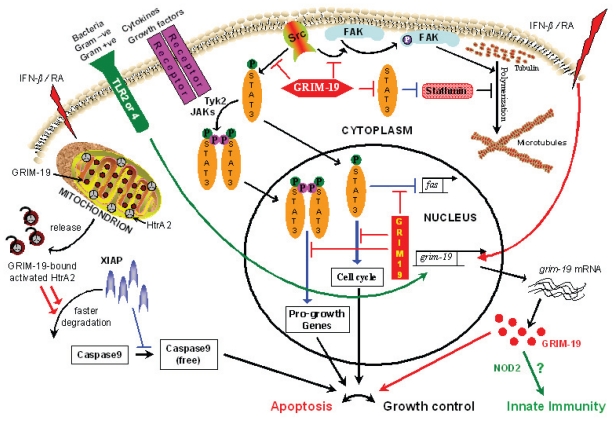
Cellular roles of GRIM-19. GRIM-19 appears to have more functions than previously envisaged: 1) It targets growth-promoting proteins, STAT3 and Src, to achieve growth control; 2) It augments the activity of HtrA2 to cleave XIAP; 3) In conjunction with NOD2 it participates in innate immune responses. GRIM-19 can perform these functions only when it is present in more than one intra-cellular compartment. For example, nuclear and cytosolic GRIM-19 can effectively block STAT3 and Src, respectively. GRIM-19 overexpression effectively blocks cell motility by decreasing tubulin polymerization. Upon IFN/RA stimulation, the *de-novo* synthesized GRIM-19 participates in growth control in conjunction with other proteins. Requirement of GRIM-19 for complex-I assembly and stability *vis-à-vis* ATP generation, is one of its reported functions. Red colored-lines and arrows represent GRIM-19-dependent inhibitory and stimulatory pathways, respectively. Blue colored lines and arrows represent STAT3-dependent inhibitory and stimulatory pathways, respectively. Green arrows represent novel GRIM-19 inductive and functional pathways. P in green and pink colored circles indicate phosphorylations at Serine 727 and tyrosine 705 residues, respectively.

**Figure 3:**
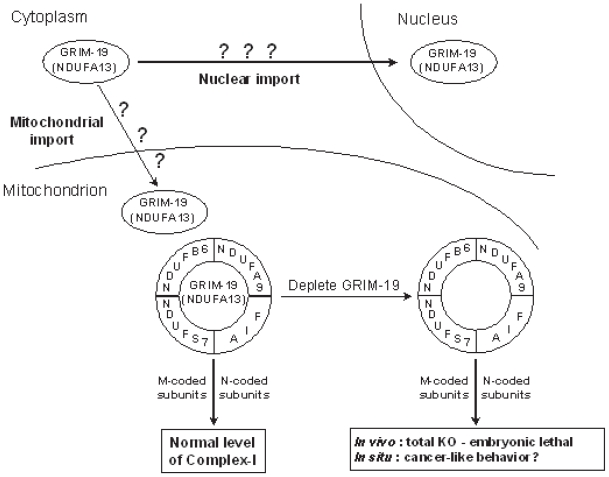
Function of GRIM-19 in steady-state. Coordinated synthesis cum assembly of mitochondrial complex-I has been well-characterized. In addition to the structural and functional proteins in complex-I, distantly-related proteins like GRIM-19 and AIF are required for proper complex-I formation. Homozygous deletion of *grim19* resulted in embryonic lethality in mice which may be a result of decreased complex-I levels in the developing embryo. M-coded—mitochondrial DNA-encoded, N-coded—Nuclear DNA-encoded.

The maintenance of genomic integrity is crucial for preventing tumor development. Cell cycle checkpoints play a crucial role in these processes. The dominant G_1_ and G_1_-S checkpoint responses are routed through p53 and pRb, which are most commonly deregulated in human cancers (Bartek et al. 1997; Kastan and Lim, 2000; Sherr and McCormick, 2002). The transcription factors E2F-1 and c-myc, targets of unphosphorylated STAT3, regulate the expression of genes associated with cell-cycle progression Cyclin D1 and Cyclin E that control G_1_-S transition (Lee et al. 2000; Ohtani et al. 1995). The ability of GRIM-19 to attenuate STAT3 responses adds a new dimension to this checkpoint. Like p53 and pRb, human tumors show loss of expression or/and mutations in GRIM-19. In this regard, GRIM-19 may now be classified as a new tumor suppressor, given a loss of its expression in tumors and accumulation of mutations in its gene in certain tumors. In S-phase, GRIM-19 blocks GW112, whose function is not known at present. The G_2_ and G_2_-M checkpoint is controlled by the activity of Cyclin B1/CDK1 kinase (Lukas et al. 1999; Onishi et al. 1997). Incidentally, Cyclin B1 also happens to be a STAT3-responsive gene that can be controlled by GRIM-19. Thus GRIM-19 may act as a ‘gentle brake’ throughout the cell-cycle in a normal cells emphasizing its role as a wide-spectrum growth controller i.e. in the absence of any stress-like situation(s). However, viruses have evolved strategies to nullify the growth-controlling function of GRIM-19 (Fig. 1). Having said this, cooperativity among GRIMs is required for full-blown apoptosis upon IFN/RA treatment that is distinct from known pathways of apoptosis and the GRIM machinery even causes cell death in p53 and Caspase 3-deficient cells (Kalvakolanu, 2004). Unlike most apoptosis-inducing proteins, whose cellular localizations are delineated, GRIM-19 appears to float inside the cell (Fig. 2) and perform its diverse house-keeping functions *viz*., apoptosis, growth-control, innate immunity, complex-I assembly *etc*. The most difficult of GRIM-19 function to study is complex-I assembly as 45-plus gene products are involved in the process and deficiency of many related gene products manifest similarly *i.e*. defective complex-I (Fig. 3). Does the sub-cellular localization of GRIM-19 determine its steady-state function and apoptotic behavior? The stress factors that activate the apoptotic behavior of GRIM-19 are currently being investigated.

## Figures and Tables

**Table 1:** Inducers of GRIM-19.

Inducer	Time	Outcome
Live bacteria and bacterial components (cell wall proteins, LPS *etc*)	2–16 h	Immunity
Ischemia	~24 h	Stress modulation
IFN-*β*- RA combination	16–48 h	Growth control

Although GRIM-19 was identified as a cell death mediator in IFN-β/RA-induced growth-suppressive effect, this gene can be induced by diverse stimuli—the fastest being by bacteria followed by ischemia and IFN-β/RA. The signaling pathway proteins involved in any of the above-mentioned pathways is not known completely.
